# Design of a Pressure Sensor Based on Optical Fiber Bragg Grating Lateral Deformation

**DOI:** 10.3390/s101211212

**Published:** 2010-12-08

**Authors:** Frantisek Urban, Jaroslav Kadlec, Radek Vlach, Radek Kuchta

**Affiliations:** 1 Department of Microelectronics, Brno University of Technology, Brno, Udolni 53, CZ-602 00 Brno, Czech Republic; E-Mails: urban@feec.vutbr.cz (F.U.); kuchtar@feec.vutbr.cz (R.K.); 2 Institute of Solid Mechanics, Mechatronics and Biomechanics, Brno University of Technology, Technicka 2896/2, CZ-616 69 Brno, Czech Republic; E-Mail: vlach.r@fme.vutbr.cz (R.V.)

**Keywords:** pressure sensor, fiber optic, fiber Bragg grating

## Abstract

This paper describes steps involved in the design and realization of a new type of pressure sensor based on the optical fiber Bragg grating. A traditional pressure sensor has very limited usage in heavy industrial environments, particularly in explosive or electromagnetically noisy environments. Utilization of optics in these environments eliminates all surrounding influences. An initial motivation for our development was the research, experimental validation, and realization of a complex smart pressure sensor based on the optical principle. The main benefit of this solution consists of increasing sensitivity, resistance to electromagnetic interference, dimensions, and potential increased accuracy.

## 1. Introduction

A pressure sensor presents key technology for the safe operation of different technical products, systems, and technologies. These sensors can be widely found in medicine as well as in different experimental, developmental, and diagnostic processes. Designing of a so-called intelligent pressure sensor has been a burgeoning trend. Intelligent pressure sensors comprise electronic circuits and passive parts, which are required, e.g., for linearization of a sensor’s characteristics and decreasing its dependence on temperature and setting of measuring range, zero point, *etc.* The most powerful microelectronic circuits enable installation of a digital pressure gauge inside a sensor and also software-control starting of different electronic responses (warning signals, *etc.*) in accordance with the measured value. Special pressure sensors exist for explosive environments. Such environments present very strict requirements for pressure sensor capsules, electronic systems (especially safety of power buses and securing these signals against unwanted failure sparkling), communication interfaces and buses to prevent possible hazard states in these extreme operating conditions.

Pressure is measured in a very wide range from 10^−12^ Pa for an extreme vacuum up to 10^12^ Pa at the research pressure of an explosion. In the widest class of pressure sensors an exactly defined deformation member is used. Its characteristic deviation, or literally deformation, is linearly related to applied pressure. This mechanical value is converted into an electrical digital signal. The method of electric conversion determines the metrological and technical characteristics of the pressure sensor.

Contemporary principles for a transmission of mechanical changes to the deformation member of electric signal are based on the piezoresistive principle [1], inductive principle [2], capacitive principle [3], piezoelectric principle [4,5], thermo-electrical principle [6], and acoustic principle [7,8].

All of these principles have a large number of technological limitations, which restrict their full utilization for pressure sensors in environments with intensive electromagnetic interference and explosion sensitivity.

## 2. Motivation

The main goal of this project was the development and practical application of a new unique method for scanning deflection of a deformation membrane. This new method is based on an optical measuring system using optical fibers. Generally, the main advantages of optically sensing principles consist of excellent pressure sensitivity, galvanic isolation of the whole sensor via optical fiber, very good accuracy of static and dynamic measuring, maximum immunity against electrical and magnetic interference, and the miniature size of the resulting pressure sensor. The attributes of pressure sensors based on the optically sensing principle also demonstrate optimal characteristics for use in explosive gas environments and in environments with high electromagnetic interference.

This new pressure sensor unfolds new possibilities for application in very special cases, e.g., military or security applications without a threat of electromagnetic tapping. Pressure sensors, in most cases, use displacement sensing of the deformation membrane, where the level of deformation depends on the applied pressure. Generally, two basic optical principles are used for displacement sensing [9]. The first is the sensing principle based on the Fabry-Perot resonator [10–14], which uses the interference effect. The second principle is based on sensing of a light reflection using the fiber Bragg grating (FBG) [15–18]. Both types of measurement principles are very sensitive and accurate for displacement measurements, what makes them ideal for pressure sensors. On the other hand, both principles require highly sophisticated construction of pressure sensing capsules and powerful measuring electronics or software algorithms. Sensors with optical principles are used for many other applications with simpler implementation. A good example of the optical sensing principle with FBG in use is temperature measurement as elaborated in [19,20]. Close to pressure sensors are optical acoustic sensors [21,22] stress [23,24] or strain [25,26] sensors.

Sensing of these physical values by optical principles is simpler than sensing pressure in industrial environments because they not necessarily fulfill all requirements for high resolution of sensing value and construction parameters, which are required for automation application. The optical principles are generally very sensitive and require the stable mechanical construction of sensing unit with parasitic effect compensation.

## 3. Sensor Design

The fiber Bragg grating is a device commonly used in telecommunications and sensor technology. Fiber gratings are formed by a periodic change of the fiber cored refractive index in direction of propagation of optical radiation. In principle, the fiber Bragg grating acts as a spectral filter that reflects particular wavelengths of light near Bragg resonance wavelength and the rest of the optical signal spectrum is being released. The Bragg resonant wavelength is given by:

(1) $$\lambda_{\text{Bragg}}=2n_{\text{eff}}\Lambda$$

where λ*_Bragg_* is the Bragg resonant wavelength, *n_eff_* is the effective refraction index, and Λ is the periodic variation of the FBG.

FBGs used in sensors mostly rely on the spectral analysis of reflected light wavelengths. The Bragg resonant wavelength is determined by various factors applied on the FBG, which affect effectively refractive index or grating periodic variation; therefore, it is an indirect measurement resulting from modifying physical or geometrical properties of the FBG. Among the affected factors we have temperature, mechanical deformation (e.g., stretching, pushing, bending, and applying shear stress) to the fiber Bragg grating. In real applications, it is difficult to separate the effects of measured and parasitic variables that affect the same parameter (e.g., when the fiber Bragg grating deformation is measured, temperature also affects reflected light wavelengths). Pressure measurement is always based on the deformation of some sensing part (typically the membrane), which is afterwards measured by principles described in the first section.

**Figure 1.**
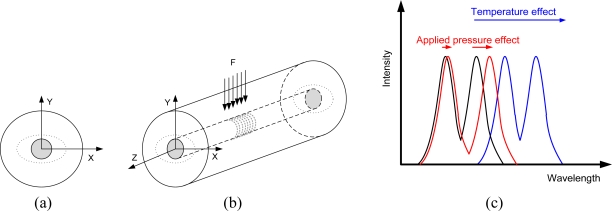
**(a)** Cross-section of the FBG with marked fiber shape (dashed line) under pressure. **(b)** Schematic drawing of the FBG with indicated direction of applied pressure. **(c)** Spectral response of the FBG depending on the applied strain and temperature.

Applied stress on the fiber Bragg grating in the direction of the fiber axis results in the extension of its physical dimensions and in the change of the periodic variation; however, the influence of temperature also affects physical dimensions due to thermal expansion. Mathematically, the shift in the Bragg wavelength λ_Bragg_ due to an applied strain and temperature change is given by [27]:

(2) $$\Delta \lambda_{\text{Bragg}}=2\left(\Lambda \frac{\partial n_{\text{eff}}}{\partial l}+n_{\text{eff}}\frac{\partial \Lambda }{\partial l}\right)\Delta l+2\left(\Lambda \frac{\partial n_{\text{eff}}}{\partial l}+n_{\text{eff}}\frac{\partial \Lambda }{\partial l}\right)\Delta T,$$

where *l* is the FBG length and *T* is temperature. Similar deformation tests with FBGs were published in [28]. FBG spectrum deformation can be also created by applying strain not only to extend a length of FBG but also by pressing FBG to get an ellipsoidal fiber cross-section shape. This deformation implies a different effect on the resulting spectrum which splits into two peaks [Figure 1(c)] where the central frequency distance is equal to the fiber cross-section deformation ratio. Based on the preconditions FBG is under a loading state of plane strain and the components of strain normal to this plane are zero (ɛ_z_ = 0) [29,30] the wavelength response of the high birefringence FBG at any point can be expressed as follows [31]:

(3) $$\Delta \lambda_{X}=\lambda_{X}\left\{-\frac{n_{0}^{2}(1+\upsilon )}{2E}[(1-\upsilon )(p_{11}\sigma_{X}+p_{12}\sigma_{Y})-\upsilon (\sigma_{X}+\sigma_{Y})]\right\}$$

(4) $$\Delta \lambda_{Y}=\lambda_{Y}\left\{-\frac{n_{0}^{2}(1+\upsilon )}{2E}[(1-\upsilon )(p_{11}\sigma_{Y}+p_{12}\sigma_{X})-\upsilon (\sigma_{X}+\sigma_{Y})]\right\}$$

where shear stress is ignored and *E* and *υ* are the Young’s modulus and Poisson’s coefficient of the optical fiber, *p*_11_ and *p*_12_ are the strain-optic coefficients, *n*_0_ is the refractive indices for the axe, *σ*_X_ and *σ*_Y_ are the stress components in the principal directions, and *λ*_X_ and *λ*_Y_ are the initial wavelengths of the peaks corresponding to the two polarization modes, Δ*λ*_x_ and Δ*λ*_Y_ are the wavelength shifts in the axes.

Various types of FBG-based sensors have been developed, *i.e.*, [25,26,32] however the majority of these tests or described pressure sensors use the longitudal FBG’s deformation principle, which results in bigger optical signal wavelength displacement (FBG central frequency moves from 10 to 30 nm) unlike our method of pressing the FBG laterally, to obtain an ellipsoidal fiber cross-section shape, which generates a maximum of 300 pm spectrum peak spread. On the other hand, measured peaks` distance is not influenced by temperature effects. Designing a pressure sensing capsule with longitudal FBG deformation and temperature compensation of all parasitic material temperature expansions including the parasitic temperature effects on the measured value is admittedly challenging, moreover for pressure sensing capsule targeted on the automation field. From this reason a lateral deformation of the FBG and more robust sensor capsule became an acceptable way for our sensor capsule design and the subsequent manufacture and assembly process. Lateral deformation of FBG by applied pressure is described in [33], but in fact lateral deformation of coating material around FBG is transformed into longitudal mechanical stretching of FBG along its axis. A crucial factor for applying pressure to FBG is uniform stress along the entire length. This was one of most important parameters during the process of designing our pressure sensing capsule.

In our newly developed sensor this parasitic temperature influence is eliminated by the proposed measurement principle and by the optional use of a second FBG as a reference. This reference FBG senses only temperature not applied stress from deformation membrane. Measured capsule temperature can be used to compensate for the thermal expansion of mechanical components in the sensing capsule and possibly to alarm users when the operating temperature exceeds allowed limits. However, this method brings additional financial costs due to necessity of a second FBG, and a more comprehensive sensor capsule mechanical solution. Temperature compensation of the FBG sensor can be solved by special thermo-package design, which is also described in [20], but in our sensing principle we have used this parasitic temperature influence to measure pressure capsule temperature. From this value we are able to compute the pressure sensing capsule’s thermal expansion and thus minimize this parasitic effect. Therefore there is no more need to solve temperature compensation of FBG sensor itself. According to theoretical expectations applied pressure and temperature indicate different effect on the output signal characteristic and do not interface each other.

**Figure 2.**
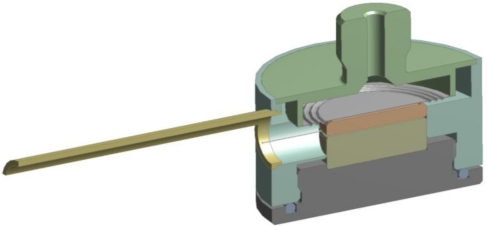
Design pressure sensor that is used for computational model, and subsequently in manufacturing process.

For the purposes of our experiment, we have designed a pressure capsule with two sensing FBGs. The primary FBG, which senses pressure, was built inside sensing capsule together with the second FBG for parasitic temperature calibration. The second temperature measurement FBG is placed inside of our sensor as well to verify homogenous temperature distribution inner pressure sensing capsule, however is not necessary for the final application. In fact, the secondary FBG doubles the temperature measuring functionality of the primary pressure sensing FBG. A general pressure capsule designed with incorporated measuring FBGs can be seen in Figure 2.

## 4. Measurement Test-Bed

**Figure 3.**
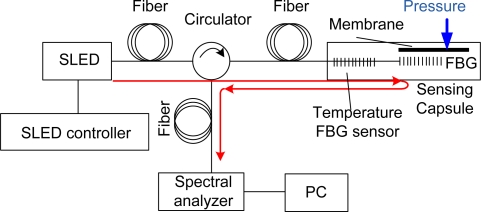
Complete measurement setup with super luminescent diode as the source of optical signals, optical path components and spectral analyzer for evaluation of reflected optical signals from the sensing FBG. The red line shows the optical signal trace and the blue arrow the direction of an applied pressure.

The measurement setup consists of a optical signal source, in this case a 1 mW fiber coupled super luminescent diode Q Photonics QSDM-1550-2 with a central wavelength of 1,535 nm and spectral width (FWHM) of 35 nm, optical path with the single mode fiber and polarization insensitive mini fiber optic circulator NS-3-131-A9 from New Span Opto-Technology Inc., our developed pressure sensing capsule with incorporated 2 cm long FBG from O/E Land Inc. with central wavelength 1,534 nm and 0.1 nm bandwidth, high resolution (resolution bandwidth FWHM65 pm) spectral analyzer EXFO FTB-5240 with connected PC for data post-processing, and precise pressure controller/calibrator PPC3-DHI from Chell Instruments Ltd. A complete diagram of the used measurement setup is given in Figure 3.

## 5. Stress Analysis of Pressure Sensors

**Figure 4.**
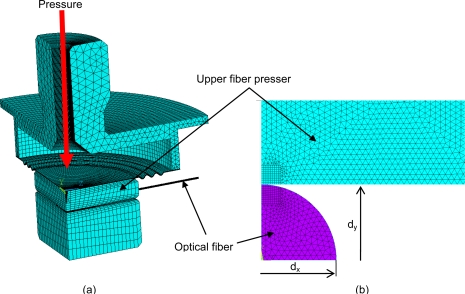
**(a)** Global and **(b)** detailed FEM computational model of pressure sensor based on the fiber Bragg grating deformation.

The aim of stress analysis was the verification of our pressure sensor design. The stress limit of the optical fiber is a crucial factor, which is a limitation of using this pressure sensing method, based on the fiber Bragg grating deformation, whereas lateral deformation of optical fibers is desired for exact operation of the pressure sensor. The mechanical design of the pressure sensor (Figure 3) was used for verification of the computational model’s (Figure 4) applicability to a pressure sensor.

The ANSYS software, which comes from the finite elements method (FEM), was used for computational modeling in [34–41]. The stress analysis was divided into two computational parts. The first part is targeted to global stress analysis. The result of the first analysis was used for the second computational simulation, which was targeted to the detailed analysis of optical fiber deformation. Properties of the used material are:

- Steel—elastic modulus 210,000 MPa
  ○ Poisson’s ratio 0.3
- Optical fiber—elastic modulus 73,000 MPa
  ○ Poisson’s ratio 0.17
  ○ Strength limit 1,100 MPa

**Figure 5.**
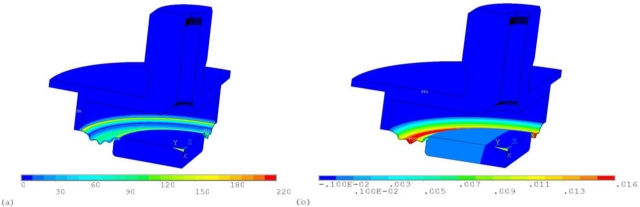
**(a)** Von Mises stress distribution and **(b)** X-direction deformation in the membrane for pressure of 2 bars.

The global stress analysis is aimed for the determination of stress and deformation of the membrane, as shown in Figure 5 and calculation of the force, respectively line load, by means of which the optical fiber is loaded. The membrane is loaded by pressure in the inner space of the sensors.

The detailed stress analysis of optical fiber deformation was made for the calculation of cross deformation, which is used for pressure evaluation using a sensing method based on the fiber Bragg grating deformation. The symmetry of geometry was also assumed, so only one quarter of the optical fiber was modeled [Figure 4(b)]. The effect of fiber-end loading was neglected, so 2D analysis was assumed.

The loading was applied to an upper fiber presser, where the value of force loading the optical fiber was determined from the previous global analysis. The simulation result is the distribution of stress and level of deformation. The cross displacement ratio of axes *d_x_* and *d_y_* is more important in terms of pressure evaluation possibilities using a sensing method based on the fiber Bragg grating deformation.

**Figure 6.**
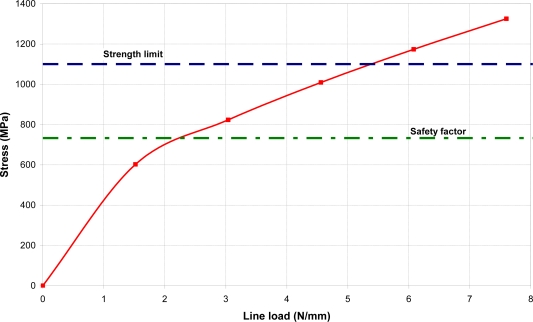
Relationship of maximal Von Mises stress in the optical fiber on an optical fiber line load with respect to a strength limit of 1,100 MPa and safety factor of 1.5.

For an optical fiber safety factor of 1.5 a maximal stress level up to 733 MPa is permitted (Figure 6). This corresponds to a line load of 2.25 N/mm. A maximal pressure of 2 bars can be applied onto the sensor, when the length of the pressed fibers is approximately 19 mm. The maximal allowed pressure value was determined using the global analysis [Figure 4(a)]. The computational simulation was solved for determination of the pressure corresponding to a line load on optical fiber of 2.25 N/mm.

The relationship of the cross displacement ratio of axes *d_x_* and *d_y_* on the line load is nearly linear; therefore, it is possible to derive the following equation from the FEM simulation results:

(5) $$R_{d}=10.88\cdot q_{l}$$

where *R_d_* is the cross displacement ratio of axes and *q_l_* is the line load on the optical fiber. Equation (5) was determined using the detailed stress analysis [Figure 4(b)], where the simulations with several line loads were calculated and the cross deformation of axes was evaluated. The maximal line load was of 2.25 N/mm, as mentioned above.

The results of the FEM simulations can be used for pressure sensor design and mainly for algorithm design of sensing methods based on Bragg grating deformation. The pressure range of sensors can be extended using longer optical fibers, which is suppressed between pressers. However limiting line load is necessary to verify this.

## 6. Results

This optical measurement principle is based on an interaction between applied pressure and mechanical deformation of the used FBG, resulting in changes of the shape of reflected spectral characteristics. The reflected spectrum is spread into two peaks whose relative position determines the level of the FBG deformation. Relations between the FBG deformation and distance of two spectral peaks of reflected light is not linear but can be described by second order polynomial function:

(6) $$f(x)=a\cdot x^{2}+b\cdot x+\lambda_{\text{Bragg}}-\frac{\sigma }{2}$$

where *a* and *b* are constant, λ*_Bragg_* is fiber Bragg grating central wavelength and is fiber Bragg grating spectral width.

**Figure 7.**
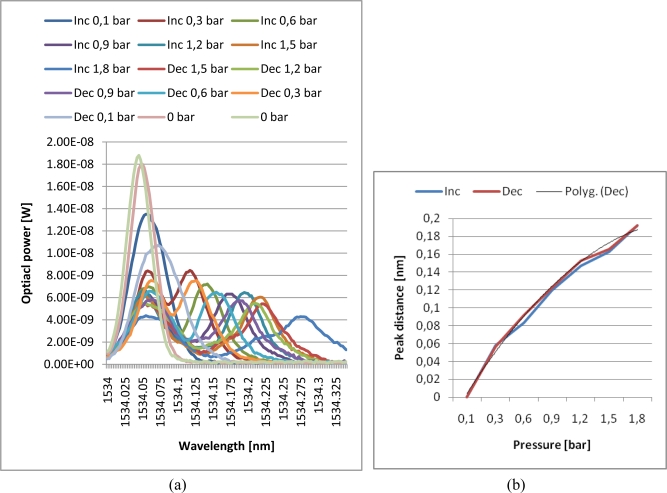
**(a)** Measured spectral characteristics from realized pressure sensor (notes: Inc—incrementing pressure, Dec—decrementing pressure). **(b)** Dependence of applying pressure on the resulted calculated peaks wavelength distance value.

Moreover, the distance of these peaks is temperature independent and carries only information of the degree of the FBG deformation. The measured characteristic matches the theoretical expectations exactly [see Figure 1(c)]. Spectral characteristics at different pressures are shown in Figure 7(a). The lowest course with the biggest distance between peaks corresponds to the maximum pressure and the course with only one and the biggest peak stands for zero pressure.

To get more precise results of the peak maximum position, measured characteristics have to be evaluated by regression analysis. Peak distance value provides information about applied pressure. Therefore a more accurate evaluation of peak position can significantly improve the precision of the whole pressure measurement process. From the physical spectrum measurement principle of the used spectral analyzer and the real measured characteristics we derived as the best course for regression analysis a Gaussian curve, which can be described by:

(7) $$f(x)=a\cdot e^{-\frac{{\left(x-b\right)}^{2}}{2c^{2}}}$$

where *a*, *b*, *c* > 0 are constants, which we are obtain from regression analysis and *x* represents single spectrum wavelength from the measured characteristics. As a mathematic core for curve fitting we implemented Levenberg–Marquardt algorithm (LMA) [42].

The disadvantage of this measurement is the requirement of a high resolution (∼10 pm) spectral analyzer for precise measurement and the need for post-processing of measured spectral characteristics by regression analysis, which is time consuming and computationally demanding. Interpolation of both measured peaks by regression analysis with Equation (7) significantly improves general pressure measurement precision but also increases the cost for final realization and the complexity of this newly developed pressure sensing system. On the other hand to get the same result without data post-processing and curve fitting it is necessary to have more precise spectral analyzer with higher resolution, below 1 pm. The used SW regression analysis can provide the same resolution with significantly lower cost.

**Figure 8.**
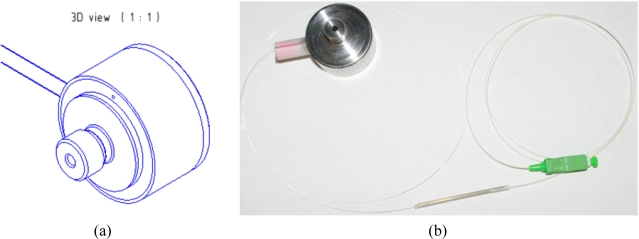
**(a)** 3D manufacturing drawing of a pressure sensor capsule **(b)** Realized pressure sensor capsule from stainless steel with second reference FBG placed outside the sensor.

Based on the designed and simulated pressure sensing capsule with lateral FBG deformation we made a prototype of this sensor [see Figure 8(a) drawing and Figure 8(b) final sensor prototype] to validate the correctness of our predictions and theoretical expectations.

The pressure measurement error was determined to be 3.13% by averaging all measurements (including increasing and decreasing pressure to also evaluate sensing membrane hysteresis). Final capsule design fulfills the main requirements for easy installation and fast calibration. Capsule and scanning principle also have excellent resistance to the parasitic effects of temperature when the measured temperature error of the sensing capsule is lower than the temperature error of the spectral analyzer used. The disadvantages of this measurement principle are high demand on the spectral analyzer resolution and strictly precise construction of the sensor capsule with a guaranteed uniform load from one direction to the Bragg grating along its whole length.

## 7. Conclusions

A new pressure sensor based on the optical principle was developed, realized, and tested. Final construction was designed with planned automation applications in mind. A basic drawing of the new pressure sensor is displayed in Figure 2. The sensing section achieves galvanic isolation of the optical fiber from the measuring unit and can be placed without risk in an explosive industrial environment. The length of the optical fiber can be up to 1 km. The parasitic influence of temperature, which causes the biggest measuring error, was eliminated by the thermo-compensation method used for the sensing capsule design and also by the software algorithm in the evaluation unit. Thermo-compensated design was first analyzed and simulated by the engineering design analysis software (Figure 4) and then tested on the prototype. Measured characteristics from Figure 7(a) are according to theoretical values from Figure 1(c) and allow simple software evaluation of measured pressure. Measured results from cyclic tests [see Figure 7(b)] show final pressure measuring precision under 3.13% over a pressure range (0–1.8 bar) in operation temperature range from −20 °C to +70 °C. Achieved parameters and the materials used make this optical pressure sensor ideal for use in industrial environments, explosive environments, and environments with high electrical and magnetic interference. The main known disadvantage in comparison to the contemporary pressure sensors is slightly worse precision and its higher price. Our future work will be focused mainly on research and development of a cheaper evaluation unit with better resolution for reaching even better measuring precision paired up with a lower error rate.
